# Pretreatment body mass index as a prognostic factor for clinical course and survival in primary surgically treated oral squamous cell carcinoma – a retrospective study

**DOI:** 10.3389/fonc.2026.1894543

**Published:** 2026-10-02

**Authors:** Eike Floegel, Leonard Simon Brandenburg, Janek Aaron Arias Romero, Kirstin Vach, Marc Christian Metzger, Rainer Schmelzeisen, Jonas Wüster

**Affiliations:** 1 Department of Oral and Maxillofacial Surgery, University Medical Center Freiburg, Faculty of Medicine, Albert-Ludwigs-University of Freiburg, Baden-Württembergm, Freiburg, Germany; 2 Institute of Medical Biometry and Statistics, University Medical Center Freiburg, Faculty of Medicine, Albert-Ludwigs-University of Freiburg, Baden-Württemberg, Freiburg, Germany; 3 Centre for Data Science, Eberswalde University for Sustainable Development, Eberswalde, Germany

**Keywords:** body mass index, free-flap reconstruction, oral squamous cell carcinoma, overall survival, recurrence-free survival, red blood cell transfusion

## Abstract

**Introduction:**

In oral squamous cell carcinoma (OSCC), body mass index (BMI) is an available but imperfect marker of nutritional reserve. Evidence on pretreatment BMI in surgery-based European OSCC cohorts remains limited, especially regarding perioperative outcomes. This study evaluated associations of pretreatment BMI with overall survival (OS), recurrence-free survival (RFS), and perioperative outcomes after curative-intent R0 resection.

**Methods:**

This retrospective single-center cohort included adult patients with previously untreated primary OSCC, available pretreatment BMI, and curative-intent R0 surgery between 2014 and 2023. BMI was assessed continuously and by World Health Organization (WHO) categories. OS and RFS were analyzed using multivariable Cox regression adjusted for age, sex, pathological T category, nodal status, microvascular free-flap reconstruction, and diabetes mellitus. Perioperative outcomes included red blood cell (RBC) transfusion, RBC units transfused, percutaneous endoscopic gastrostomy (PEG) placement, and intensive care unit (ICU) and hospital length of stay.

**Results:**

A total of 274 patients with available pretreatment BMI were included; 116 deaths occurred. Five-year OS ranged from 31.4% in patients classified as underweight to 82.0% in patients with obesity. Higher BMI was associated with lower mortality (HR per 1 kg/m² 0.95, 95% CI 0.91–0.99; p = 0.013), and obesity was associated with improved OS compared with normal weight (HR 0.50, 95% CI 0.26–0.96; p = 0.037). BMI was not significantly associated with RFS after adjustment (HR per 1 kg/m² 0.97, 95% CI 0.93–1.00; p = 0.080). Higher BMI was associated with fewer perioperative RBC transfusions (any transfusion: OR 0.91, 95% CI 0.84–0.98; p = 0.012; number of units: OR 0.89, 95% CI 0.82–0.95; p = 0.001). BMI was not independently associated with PEG placement or hospital length of stay; ICU findings depended on BMI modeling.

**Discussion:**

Higher pretreatment BMI was associated with better OS and lower perioperative RBC transfusion requirements, whereas associations with RFS and most other perioperative outcomes were not significant after adjustment. Given the retrospective design and limitations of BMI as a body-composition marker, these findings should be interpreted cautiously. Pretreatment BMI may help identify low-BMI patients warranting early supportive-care assessment.

## 1 Introduction

Oral squamous cell carcinoma (OSCC) is the most common malignant tumor of the oral cavity worldwide and is among the most frequent malignancies of the head and neck (1–3). Globally, cancers of the lip and oral cavity account for approximately 389,485 incident cases and 188,230 deaths per year, highlighting the substantial global burden of these cancers (4). Despite advances in surgery, radiotherapy, and systemic therapy, long-term survival has improved only modestly (5). Although perioperative PD-1-directed immunotherapy has expanded treatment options in resectable locally advanced head and neck cancer (HNC), curative-intent management of OSCC remains centered on surgery (6). Based on SEER data, five-year relative survival is approximately 86% when diagnosed at a localized stage, declining to around 69% once regional spread is present (7, 8). Across large retrospective cohort studies, 5-year overall survival (OS) commonly ranges from 40% to the mid-50% range, even with surgery-based multimodal management (9–11). Accordingly, nodal involvement remains one of the most important predictors of survival, alongside tumor stage and other clinicopathologic factors (12, 13). Retrospective analyses have also linked outcomes to commonly used medications, supporting evaluation of pretreatment body mass index (BMI) as a pragmatic nutritional marker (14–16).

BMI is a simple, routinely recorded anthropometric measure used to categorize adult weight status (2, 17). In oncology, BMI has been evaluated not only in relation to cancer risk but also as a prognostic marker after diagnosis, with heterogeneous associations across tumor entities and settings (18, 19). Notably, some cohorts report improved outcomes in overweight or mildly obese patients (“obesity paradox”), while underweight is more consistently linked to poorer survival (19, 20).

In HNC and OSCC specifically, available evidence on pretreatment BMI and survival remains heterogeneous and largely derives from East Asian cohorts or selected single-institution North American series focusing on specific stages or treatment concepts (2, 3, 19, 21–24). There is limited evidence from contemporary Central European, surgery-based OSCC cohorts that evaluate pretreatment BMI alongside comorbidities and detailed clinicopathologic risk factors. While a recent large German single-center cohort reported an independent association between pretreatment underweight and poorer survival, evidence from European cohorts remains limited, and the generalizability of findings from predominantly non-European populations is uncertain (22, 25). As a routinely captured measure, BMI may provide pragmatic prognostic information to support risk stratification and identify patients who could benefit from early supportive-care interventions (2, 26, 27). Beyond survival outcomes, the impact of pretreatment BMI on the perioperative clinical course in surgery-based OSCC cohorts remains less well defined. We therefore assessed the association of pretreatment BMI with OS and recurrence-free survival (RFS) and examined its relationship with key perioperative endpoints, including hospital and intensive care unit (ICU) length of stay (LOS), red blood cell (RBC) transfusion requirements, and percutaneous endoscopic gastrostomy (PEG) placement, in a retrospective European tertiary-care cohort of previously untreated OSCC patients undergoing primary curative-intent R0 surgery.

## 2 Materials and methods

### 2.1 Ethics statement

This study was approved by the ethics committee of the Albert-Ludwigs-University Freiburg, Germany (No. 25-1222_1-S1-retro, 16 December 2025) and was performed in accordance with the Declaration of Helsinki as revised in 2013.

### 2.2 Patient consent statement

In accordance with the statement of the ethics committee, individual consent of the patients was not necessary as the data were processed retrospectively after anonymization.

### 2.3 Inclusion and exclusion criteria

All patients of legal age with histologically confirmed primary, previously untreated OSCC who underwent primary curative-intent resection at the Department of Oral and Maxillofacial Surgery, University Medical Center Freiburg, Germany, between 1 January 2014 and 31 December 2023 and had available pretreatment BMI in the preoperative records were eligible for inclusion in the final analytic cohort. Patients were excluded if they presented with recurrent or previously treated disease, underwent salvage surgery after primary treatment elsewhere, had non-squamous malignancies, had positive resection margins (R1/R2), or had insufficient perioperative records.

### 2.4 Data acquisition

Clinical data were retrieved retrospectively from electronic medical records. The index date was the date of primary surgery. Vital status and date of death were obtained from the regional cancer registry, whereas recurrence was assessed from institutional follow-up documentation. Pretreatment BMI (kg/m²) was extracted from preoperative records. BMI categories followed the World Health Organization (WHO) classification (underweight <18.5; normal weight 18.5–24.9; overweight/pre-obesity 25.0–29.9; obesity class I 30.0–34.9; class II 35.0–39.9; class III ≥40.0 kg/m²) (28, 29). Perioperative variables during the index hospitalization were extracted from perioperative documentation, including ASA class, RBC transfusion (any; number of units), PEG placement (yes/no), hospital LOS, ICU LOS, and diabetes mellitus status at surgery (yes/no). No distinction was made between the different types of diabetes mellitus. Treatment followed contemporary institutional standards and guideline-based recommendations as determined by the multidisciplinary tumor board (27).

### 2.5 Statistical analysis

OS served as the primary endpoint and analyses were conducted as complete-case analyses. Patients with missing outcome/time-to-event information or missing covariates were excluded from the respective models, resulting in model-specific sample sizes. Pretreatment BMI was analyzed both as a continuous measure (per 1 kg/m² increase) and as a categorical exposure according to the WHO classification. For categorical analyses, obesity classes I–III were pooled (BMI ≥30.0 kg/m²) to ensure sufficiently large group sizes. Normal weight served as the reference category in categorical BMI models. Kaplan–Meier methods were used to estimate survival functions for both OS and RFS. Univariable and multivariable Cox regression models were used to evaluate associations with OS and RFS. The proportional hazards assumption was assessed using Schoenfeld residuals. Multivariable models included age, sex, pathological tumor stage (pT), pathological nodal status (pN), microvascular free-flap reconstruction, and diabetes. Age at surgery was modeled as a continuous variable, with effect estimates referring to a one-year increase; sex, pathological nodal status, microvascular free-flap reconstruction, and diabetes mellitus were modeled as categorical variables, while pathological tumor stage was entered categorically with pT1 as the reference category. Biological sex as documented in the medical records was included as a covariate; gender-related variables were not available in this retrospective dataset. Results were reported using HRs with 95% confidence intervals. For subsequent pairwise comparisons, the method of Scheffé was used to correct for multiple testing. Binary perioperative endpoints (any RBC transfusion and PEG placement) were evaluated using multivariable logistic models to estimate ORs with 95% confidence intervals. The number of transfused RBC units (modeled as ordered categories) was analyzed using multivariable ordinal logistic regression, reporting ORs with 95% confidence intervals. Count outcomes (ICU LOS and hospital LOS, in days) were modeled using multivariable Poisson regression to estimate incidence rate ratios (IRRs) with 95% confidence intervals. ICU LOS was analyzed among ICU-admitted patients only. Statistical significance was set at p < 0.05. Stata 19 (StataCorp LLC, Texas, USA) was used for statistical analysis.

## 3 Results

### 3.1 Study group characteristics

Of 509 patients reviewed for oral cancer surgery at our department between 2014 and 2023, 74 were excluded because of non-squamous histology or recurrent/previously treated disease, and a further 161 were excluded because of R1/R2 resection margins or insufficient perioperative records, yielding a final analytic cohort of 274 patients (**Figure 1**). Mean pretreatment BMI was 25.1 ± 5.0 kg/m². Within the final analytic cohort, 17 patients (6.2%) were underweight, 130 (47.5%) normal weight, 81 (29.6%) overweight, and 46 (16.8%) obese. The sex distribution in the final analytic cohort was 150 men (54.7%) and 124 women (45.3%) (**Supplementary Figure S1**). Mean age (± SD) at surgery was 64.6 ± 12.4 years, and mean follow-up (± SD) was 58 ± 36.6 months (**Table 1**). During follow-up, 116 deaths occurred. Among included patients, 159 (58.0%) underwent microvascular free-flap reconstruction. Pathologic tumor stage was available for 274 patients and broadly distributed, with pT2 being most frequent [pT1 n = 70 (25.6%), pT2 n = 89 (32.5%), pT3 n = 56 (20.4%), pT4 n = 59 (21.5%)]. Nodal status was pN0 in 173 (63.1%), pN1 in 40 (14.6%), pN2 in 45 (16.4%), and pN3 in 16 (5.8%) patients. Histopathologic grading (n = 271) was predominantly G2 [n = 202 (74.5%)], followed by G3 [n = 48 (17.7%)], G1 [n = 17 (6.3%)], and G4 [n = 4 (1.5%)]. Lymphovascular invasion (LVI) was present in 76 patients (27.7%), whereas vascular invasion was rare (6; 2.2%). Behavioral risk-factor data were available for 273 patients; 96 (35.2%) had none documented, 21 (7.7%) alcohol only, 77 (28.2%) nicotine only, and 79 (28.9%) combined alcohol and nicotine exposure (**Table 1**). When stratified by BMI category, underweight patients disproportionately presented with advanced disease features, including higher rates of pT4 tumors (41.2%), nodal positivity (64.7%), and LVI (58.8%) compared to all other BMI strata (**Table 1**).

**Figure 1:**
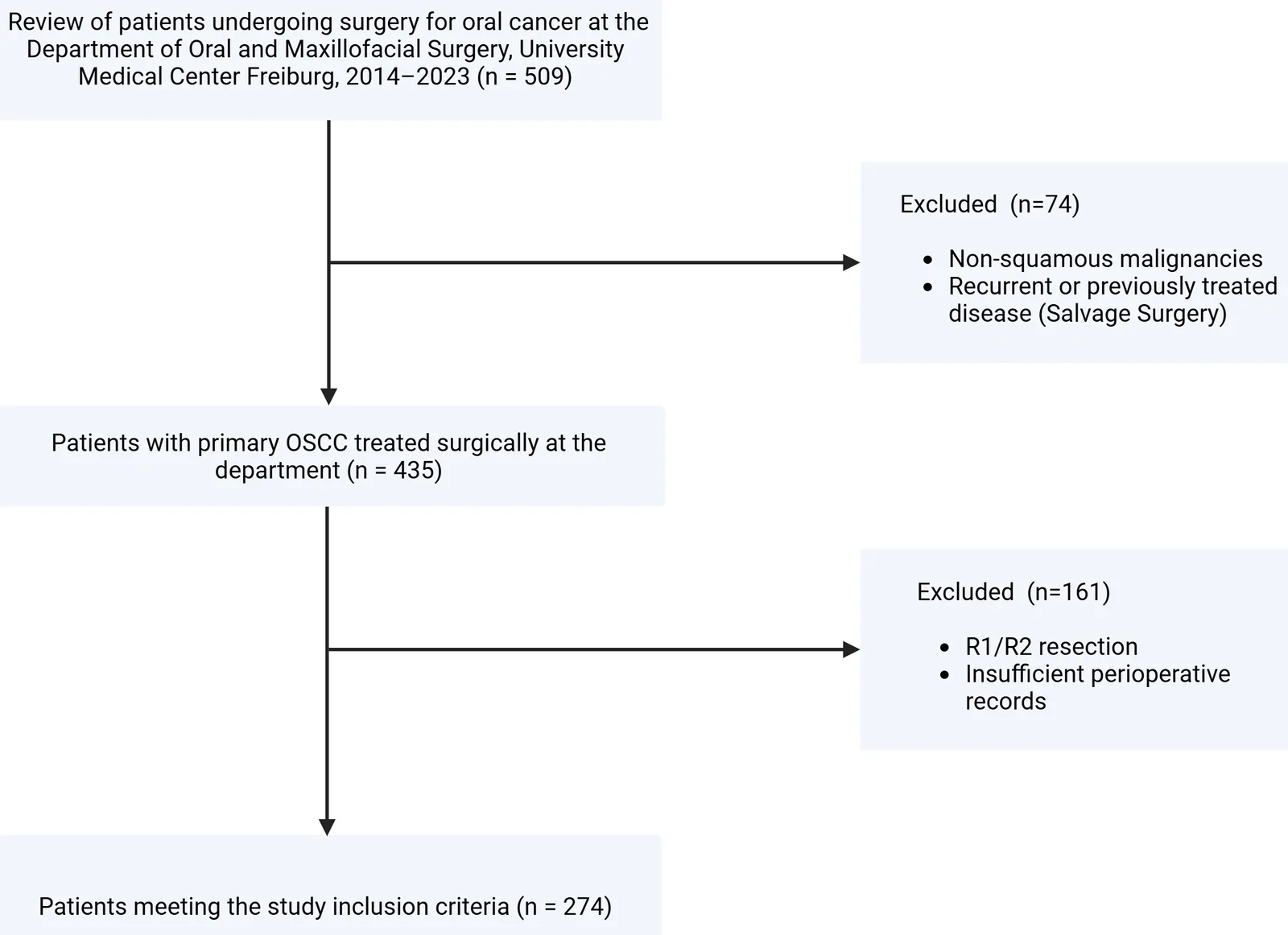
Flowchart of patient selection.

**Table 1:** Demographic, clinicopathologic, and treatment characteristics stratified by pretreatment BMI category.

Characteristic	Underweight (n = 17)		Normal weight (n = 130)		Overweight (n = 81)		Obesity (n = 46)		Total (N = 274)	
	N	%	N	%	N	%	N	%	N	%
Age at surgery (years)										
<50	0	0	17	13.08	6	7.41	4	8.7	27	9.85
50–59	5	29.41	27	20.77	17	20.99	10	21.74	59	21.53
60–69	5	29.41	49	37.69	33	40.74	13	28.26	100	36.5
70–80	2	11.76	21	16.15	18	22.22	13	28.26	54	19.71
> 80	5	29.41	16	12.31	7	8.64	6	13.04	34	12.41
Sex										
Female	11	64.71	62	47.69	29	35.8	22	47.83	124	45.26
Male	6	35.29	68	52.31	52	64.2	24	52.17	150	54.74
ASA classification										
ASA I	0	0.00	5	3.85	1	1.23	1	2.22	7	2.56
ASA II	5	29.41	42	32.31	32	39.51	15	33.33	94	34.43
ASA III	11	64.71	79	60.77	46	56.79	27	60.00	163	59.71
ASA IV	1	5.88	4	3.08	2	2.47	2	4.44	9	3.30
Risk factors										
None	6	35.29	40	30.77	26	32.5	24	52.17	96	35.16
Alcohol	0	0	10	7.69	9	11.25	2	4.35	21	7.69
Nicotine	6	35.29	36	27.69	23	28.75	12	26.09	77	28.21
Alcohol + Nicotine	5	29.41	44	33.85	22	27.5	8	17.39	79	28.94
pT category										
pT1	2	11.76	34	26.15	21	25.93	13	28.26	70	25.55
pT2	4	23.53	43	33.08	26	32.1	16	34.78	89	32.48
pT3	4	23.53	24	18.46	19	23.46	9	19.57	56	20.44
pT4	7	41.18	29	22.31	15	18.52	8	17.39	59	21.53
pN category										
pN0	6	35.29	88	67.69	48	59.26	31	67.39	173	63.14
pN1	2	11.76	22	16.92	11	13.58	5	10.87	40	14.6
pN2	6	35.29	14	10.77	17	20.99	8	17.39	45	16.42
pN3	3	17.65	6	4.62	5	6.17	2	4.35	16	5.84
pN Stage (binary)										
pN+	11	64.71	42	32.31	33	40.74	15	32.61	101	36.86
pN-	6	35.29	88	67.69	48	59.26	31	67.39	173	63.14
Grading										
G1	0	0	5	3.91	10	12.5	2	4.35	17	6.27
G2	14	82.35	93	72.66	58	72.5	37	80.43	202	74.54
G3	3	17.65	27	21.09	11	13.75	7	15.22	48	17.71
G4	0	0	3	2.34	1	1.25	0	0	4	1.48
LVI										
Yes	10	58.82	29	22.31	25	30.86	12	26.09	76	27.74
No	7	41.18	101	77.69	56	69.14	34	73.91	198	72.26
VVI										
Yes	0	0	4	3.08	1	1.23	1	2.17	6	2.19
No	17	100	126	96.92	80	98.77	45	97.83	268	97.81
Free-flap reconstruction										
Yes	9	52.94	83	63.85	45	55.56	22	47.83	159	58.03
No	8	47.06	47	36.15	36	44.44	24	52.17	115	41.97
Diabetes mellitus										
Yes	3	17.65	13	10.00	17	20.99	13	28.26	46	16.79
No	14	82.35	117	90.00	64	79.01	33	71.74	228	83.21

Characteristics are shown according to WHO BMI categories (underweight <18.5 kg/m²; normal weight 18.5–24.9 kg/m²; overweight 25.0–29.9 kg/m²; obesity ≥30.0 kg/m²). Values are presented as n (%), with percentages calculated within BMI strata. The final analytic cohort comprised 274 patients; variable-specific denominators may differ due to missing data. pT and pN indicate pathological tumor and nodal categories. LVI, lymphovascular invasion; VVI, vascular invasion.

### 3.2 Overall survival

Kaplan–Meier curves showed a separation in OS across WHO BMI categories (**Figure 2**). Five-year OS was lowest in underweight and highest in obesity (underweight: 31.4%, 95% CI 10.9–54.5%; normal weight: 67.4%, 95% CI 58.2–75.0%; overweight: 62.5%, 95% CI 50.7–72.3%; obesity: 82.0%, 95% CI 67.2–90.6%). Ten-year OS was 42.8% (95% CI 30.1–54.9%) in normal-weight patients, 40.7% (95% CI 26.2–54.8%) in overweight patients, and 70.1% (95% CI 51.1–82.8%) in patients with obesity; 10-year OS was not estimable in the underweight group. Kaplan–Meier curves for overall survival stratified by age, pT stage, and pN stage are provided in **Supplementary Figures S2**–**S4**. In univariable Cox regression (reference: normal weight), BMI categories showed significant differences (p = 0.021), with underweight associated with worse OS (HR 1.96, 95% CI 1.03–3.77; p = 0.042), while overweight (HR 1.14, 95% CI 0.76–1.73; p = 0.526) and obesity (HR 0.55, 95% CI 0.30–1.04; p = 0.065) were not significant. When modeled continuously, higher BMI remained significantly associated with improved OS (HR per 1 kg/m² 0.95, 95% CI 0.92–0.99; p = 0.015). Further univariable predictors of worse OS are detailed in **Supplementary Table S1**. In multivariable Cox regression (N = 274; deaths = 116), obesity was significantly associated with improved OS compared with normal weight (HR 0.50, 95% CI 0.26–0.96; p = 0.037), while the overall BMI category effect was not significant (p = 0.111) (**Table 2**). When BMI was modeled continuously, the association persisted (HR per 1 kg/m² 0.95, 95% CI 0.91–0.99; p = 0.013) (**Table 2**). Advanced pT stage, nodal positivity, and diabetes mellitus remained significant independent predictors of worse OS, whereas age showed a non-significant trend in both models (p = 0.067 in the categorical BMI model; p = 0.056 in the continuous BMI model) and sex as well as free-flap reconstruction were not significant (**Table 2**).

**Figure 2:**
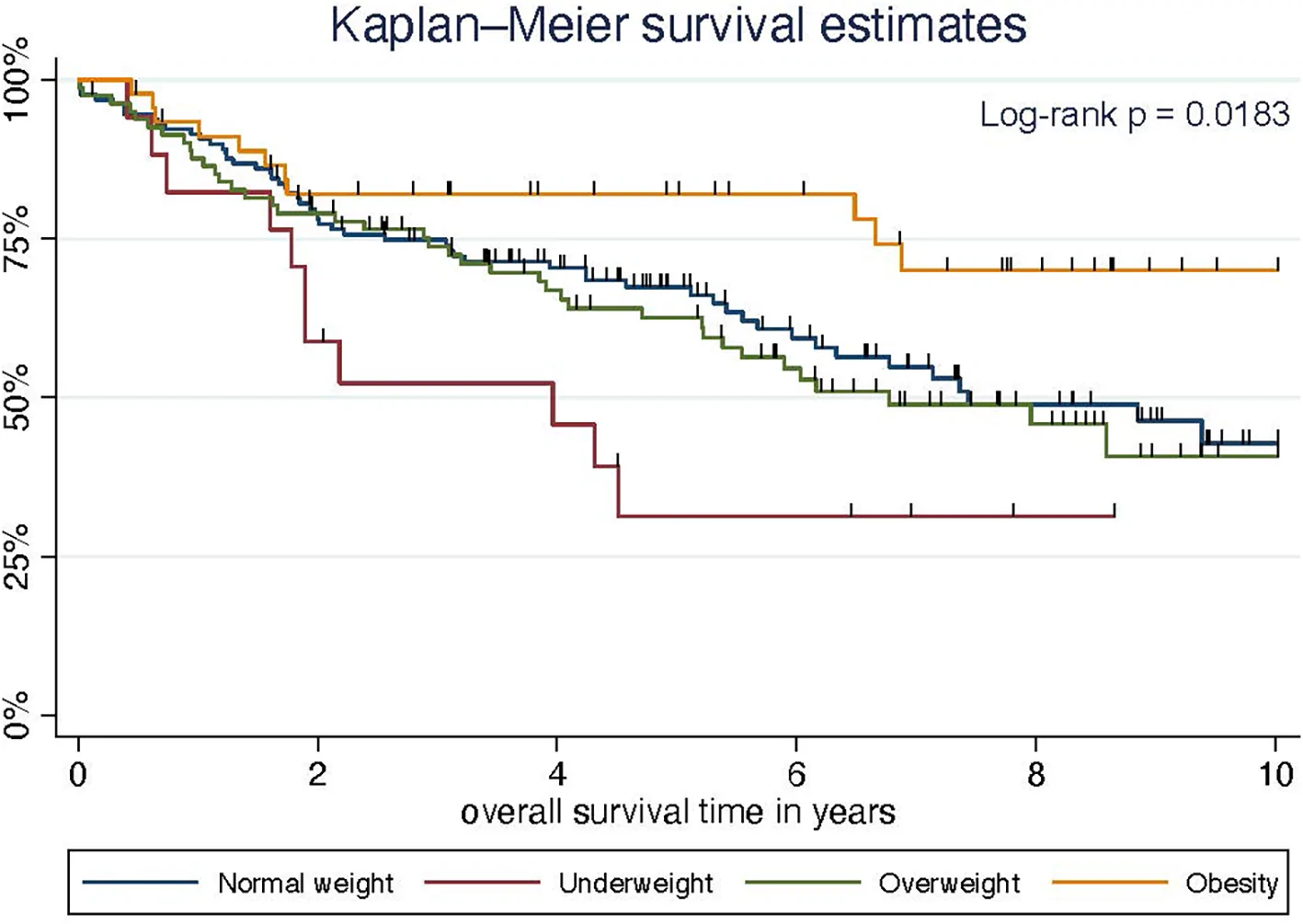
Kaplan–Meier curves for overall survival stratified by pretreatment BMI category.

**Table 2:** Multivariable Cox regression analyses for OS and RFS with BMI modeled categorically and continuously.

BMI modeled categorically	OS			RFS		
Variable	HR	95% CI	P-value	HR	95% CI	P-value
BMI						
Normal weight	1			1		
Underweight	1.35	0.69–2.65	0.378	1.28	0.68–2.40	0.438
Overweight	0.98	0.64–1.49	0.911	1.03	0.69–1.52	0.893
Obesity	0.50	0.26–0.96	**0.037**	0.69	0.40–1.17	0.168
Sex						
Male	1			1		
Female	1.15	0.78–1.71	0.475	1.04	0.73–1.49	0.813
Age						
per 1-year increase	1.02	1.00–1.03	0.067	1.01	1.00–1.03	0.128
pT Stage						
T1	1			1		
T2	1.87	1.02–3.44	**0.044**	1.50	0.91–2.48	0.112
T3	2.69	1.35–5.35	**0.005**	1.55	0.84–2.84	0.160
T4	2.49	1.25–4.95	**0.010**	1.48	0.80–2.73	0.212
pN Stage (binary)						
N0	1			1		
N+	1.49	1.01–2.20	**0.043**	1.47	1.03–2.09	**0.034**
Free-flap Reconstruction						
No	1			1		
Yes	1.21	0.76–1.93	0.413	1.33	0.87–2.02	0.185
Diabetes mellitus						
No	1			1		
Yes	1.74	1.10–2.74	**0.017**	1.43	0.94–2.19	0.093
BMI modeled continuously	OS			RFS		
	HR	95% CI	P-value	HR	95% CI	P-value
BMI						
BMI per +1 kg/m²	0.95	0.91–0.99	**0.013**	0.97	0.93–1.00	0.080
Sex						
Male	1			1		
Female	1.20	0.81–1.78	0.367	1.07	0.75–1.53	0.710
Age						
per 1-year increase	1.02	1.00–1.03	0.056	1.01	1.00–1.03	0.124
pT Stage						
T1	1			1		
T2	1.86	1.01–3.41	**0.045**	1.50	0.91–2.48	0.110
T3	2.64	1.33–5.26	**0.006**	1.53	0.84–2.81	0.166
T4	2.46	1.24–4.89	**0.010**	1.47	0.80–2.69	0.212
pN Stage (binary)						
N0	1			1		
N+	1.49	1.01–2.20	**0.042**	1.48	1.04–2.11	**0.028**
Free-flap Reconstruction						
No	1			1		
Yes	1.21	0.76–1.93	0.413	1.32	0.87–2.00	0.193
Diabetes mellitus						
No	1			1		
Yes	1.85	1.17–2.92	**0.008**	1.52	1.00–2.32	0.052

Models are reported as hazard ratios (HRs) with 95% confidence intervals (CIs) and P-values. BMI categories follow WHO definitions; normal weight was the reference category in categorical models. All models were adjusted for sex, age at surgery, pT stage, pN stage, free-flap reconstruction, and diabetes mellitus. OS, overall survival; RFS, recurrence-free survival; BMI, body mass index. Bold values indicate statistical significance.

### 3.3 Recurrence-free survival

Kaplan–Meier estimates suggested differences in RFS across WHO BMI categories (**Figure 3**). Five-year RFS was lowest in underweight (25.9%, 95% CI 8.1–48.3%) and higher in normal weight (56.1%, 95% CI 46.6–64.5%), overweight (50.7%, 95% CI 38.8–61.5%), and obesity (62.5%, 95% CI 46.1–75.3%), again with the widest confidence intervals in the underweight group. Ten-year RFS was 34.3% (95% CI 21.9–47.1%) in normal-weight patients, 36.5% (95% CI 24.3–48.8%) in overweight patients, and 58.9% (95% CI 41.8–72.5%) in patients with obesity; 10-year RFS was not estimable in the underweight group. Additional Kaplan–Meier curves for RFS stratified by age, pT stage, and pN stage are shown in **Supplementary Figures S5**–**S7**. In univariable Cox regression analysis (reference category: normal weight), BMI category was not significant overall (p = 0.117). None of the individual BMI category comparisons was statistically significant (underweight: HR 1.62, 95% CI 0.89–2.95; p = 0.112; overweight: HR 1.12, 95% CI 0.76–1.64; p = 0.558; obesity: HR 0.69, 95% CI 0.41–1.17; p = 0.166). BMI modeled continuously suggested an association toward improved RFS (HR per 1 kg/m² 0.97, 95% CI 0.93–1.00; p = 0.053). Further univariable predictors of worse RFS are detailed in **Supplementary Table S1**. In multivariable Cox regression, BMI was not associated with RFS in the categorical model (p = 0.372), and only a non-significant trend was observed when BMI was modeled continuously (HR per 1 kg/m² 0.97, 95% CI 0.93–1.00; p = 0.080) (**Table 2**). Nodal positivity remained significantly associated with worse RFS in both adjusted models (**Table 2**). Diabetes mellitus was not significant in the categorical model (p = 0.093) with a non-significant trend in the continuous model (p = 0.052), whereas pT stage and free-flap reconstruction were not independently associated with RFS after adjustment (**Table 2**).

**Figure 3:**
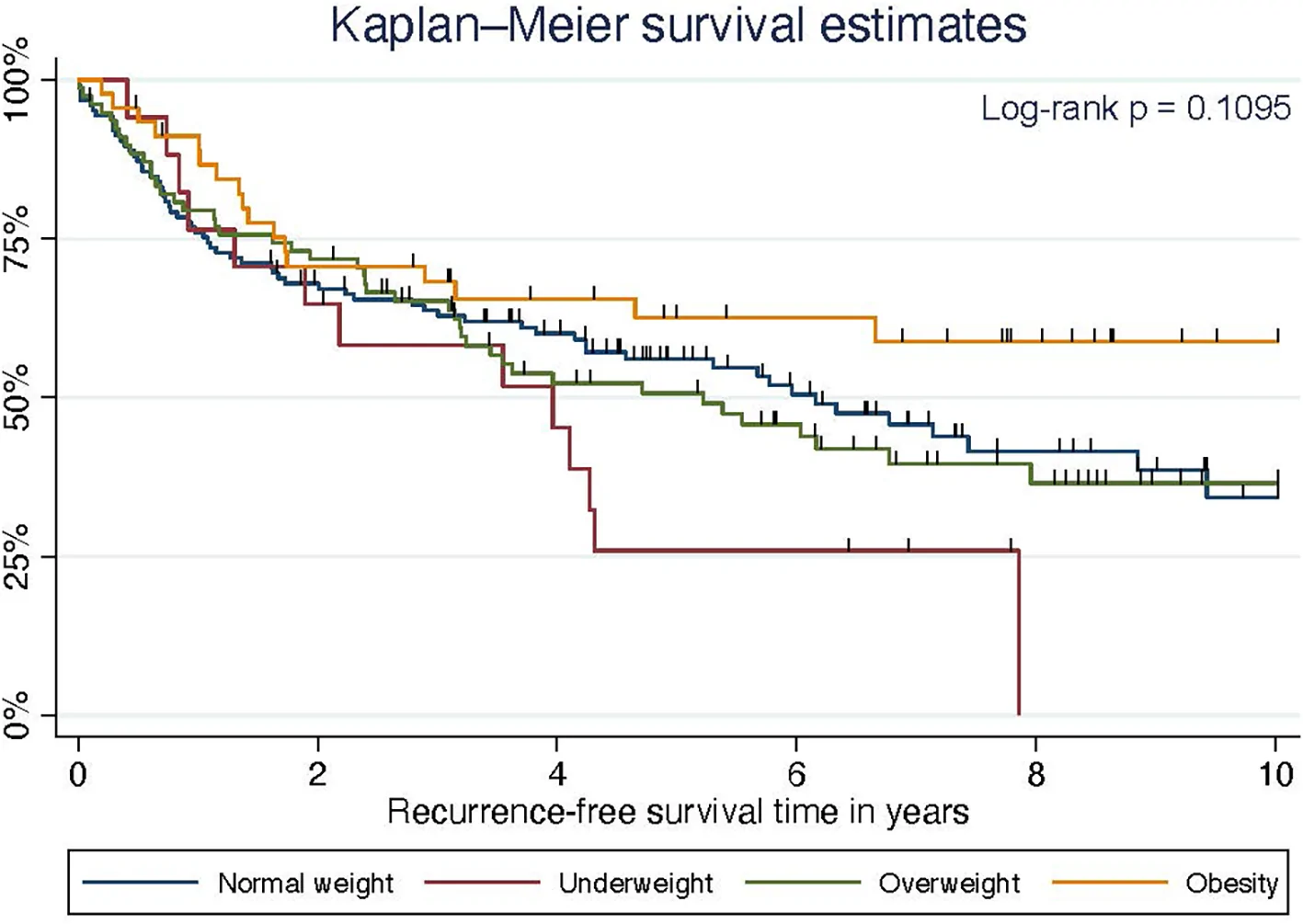
Kaplan–Meier curves for recurrence-free survival stratified by pretreatment BMI category.

### 3.4 Hospital and ICU length of stay

Hospital LOS data were available for 271 patients and hospital LOS was longer in underweight than in obese patients (23.1 ± 15.8 vs. 19.3 ± 13.1 days). In multivariable Poisson regression, no association of BMI with hospital LOS could be found, neither in the categorical model nor when modeled continuously (**Table 3**). In contrast, hospital LOS was significantly increased by free-flap reconstruction, advanced pT stage, and nodal positivity, while female sex was associated with a significantly shorter LOS (**Supplementary Table S2**). Age was linked to a small but significant increase per year (p < 0.001) and diabetes mellitus was not found to be significant (**Supplementary Table S2**).

**Table 3:** Association of pretreatment BMI with perioperative outcomes in multivariable models.

Outcome	Effect measure	Estimate	95% CI	P-value
Any RBC transfusion				
Normal weight	OR	1		
Underweight	OR	0.66	0.14–3.05	0.591
Overweight	OR	0.62	0.28–1.33	0.218
Obesity	OR	0.18	0.06–0.57	**0.004**
BMI per +1 kg/m²	OR	0.91	0.84–0.98	**0.012**
Number of RBC units transfused				
Normal weight	OR	1		
Underweight	OR	0.98	0.26–3.74	0.979
Overweight	OR	0.57	0.28–1.14	0.114
Obesity	OR	0.17	0.06–0.53	**0.002**
BMI per +1 kg/m²	OR	0.89	0.82–0.95	**0.001**
PEG placement				
Normal weight	OR	1		
Underweight	OR	2.57	0.64–10.28	0.181
Overweight	OR	0.74	0.31–1.77	0.503
Obesity	OR	1.30	0.45–3.79	0.627
BMI per +1 kg/m²	OR	0.95	0.88–1.03	0.197
ICU length of stay (days), among ICU-admitted patients				
Normal weight	IRR	1		
Underweight	IRR	1.26	0.97–1.63	0.079
Overweight	IRR	0.90	0.77–1.06	0.204
Obesity	IRR	1.13	0.94–1.37	0.184
BMI per +1 kg/m²	IRR	0.99	0.98–1.01	0.460
Hospital length of stay (days)				
Normal weight	IRR	1		
Underweight	IRR	1.01	0.90–1.12	0.911
Overweight	IRR	0.97	0.91–1.04	0.374
Obesity	IRR	0.99	0.91–1.07	0.728
BMI per +1 kg/m²	IRR	1.00	0.99–1.00	0.345

Logistic regression was used for any RBC transfusion and PEG placement, ordinal logistic regression for the number of RBC units transfused, and Poisson regression for ICU and hospital LOS. Results are reported as odds ratios (ORs) or incidence rate ratios (IRRs) with 95% confidence intervals (CIs) and P-values. BMI was modeled categorically according to WHO categories and continuously per +1 kg/m². Normal weight was the reference category in categorical BMI models. All models were adjusted for sex, age, pT stage, pN stage, free-flap reconstruction, and diabetes mellitus. RBC, red blood cell; PEG, percutaneous endoscopic gastrostomy; ICU, intensive care unit; LOS, length of stay. Bold values indicate statistical significance at p < 0.05.

Among ICU-admitted patients (n = 260), BMI showed a significant overall association with ICU LOS in the categorical model (p = 0.036), although none of the individual BMI categories differed significantly from normal weight (underweight: IRR 1.26, 95% CI 0.97–1.63; p = 0.079; overweight: IRR 0.90, 95% CI 0.77–1.06; p = 0.204; obesity: IRR 1.13, 95% CI 0.94–1.37; p = 0.184). BMI modeled continuously was not significantly associated with ICU LOS (IRR 0.99, 95% CI 0.98–1.01; p = 0.460) (**Table 3**). ICU LOS was significantly higher with microvascular free-flap reconstruction, pT4 disease, female sex, older age, and diabetes mellitus, whereas there was no significant association for nodal positivity (**Supplementary Table S2**).

### 3.5 Perioperative red blood cell transfusions

Transfusion data were available for 274 patients. With BMI modeled continuously, higher BMI was significantly associated with lower odds of any RBC transfusion (OR per 1 kg/m² 0.91, 95% CI 0.84–0.98; p = 0.012) and with fewer RBC units transfused in the proportional-odds model (OR per 1 kg/m² 0.89, 95% CI 0.82–0.95; p = 0.001) (**Table 3**). In categorical analyses (reference: normal weight), the overall BMI category effect was significant for both any RBC transfusion (p = 0.035) and the number of RBC units transfused (p = 0.017). Obesity was significantly associated with lower odds of any RBC transfusion (OR 0.18, 95% CI 0.06–0.57; p = 0.004) and of a higher transfusion category (OR 0.17, 95% CI 0.06–0.53; p = 0.002) (**Table 3**). Across transfusion models, free-flap reconstruction was the dominant significant predictor, with additional significant associations for nodal positivity. Diabetes mellitus was significant for the number of RBC units transfused but not for any transfusion. pT4 was significantly associated with any transfusion in the categorical model and showed a trend toward association in the continuous any-transfusion model as well as in the categorical ordinal model for the number of RBC units (**Supplementary Table S3**).

### 3.6 PEG placement

Data on PEG placement during the index hospitalization were available for 274 patients. In multivariable logistic regression, no independent association of BMI with PEG placement was observed, neither in the categorical model nor when BMI was modeled continuously (**Table 3**). In contrast, PEG placement was significantly associated with advanced pT stage (pT3/pT4) and free-flap reconstruction (**Supplementary Table S4**). Diabetes mellitus showed a trend toward inverse association in the categorical model, whereas sex, age, and nodal positivity were not significant predictors (**Supplementary Table S4**).

## 4 Discussion

In this retrospective single-center cohort of patients with primary R0-resected OSCC, higher pretreatment BMI was independently associated with improved OS, whereas only a non-significant protective trend remained for RFS after multivariable adjustment. In addition to oncologic outcomes, clinically relevant perioperative endpoints were evaluated. BMI was not independently associated with hospital length of stay or PEG placement after adjustment, while findings for ICU length of stay depended on BMI modeling. In contrast, transfusion-related outcomes showed an independent inverse association with BMI, with higher BMI linked to fewer RBC transfusions. Associations between BMI and post-diagnosis survival vary across cancer entities and clinical contexts, with overweight and mild obesity linked to improved survival in some settings but poorer outcomes in others (20, 30, 31). These findings are often discussed in the context of the “obesity paradox,” but remain debated due to potential bias in observational studies and the limited ability of BMI to capture body composition (32, 33). In HNC and OSCC specifically, available evidence remains heterogeneous; however, many cohorts report that low pretreatment BMI, particularly underweight, is associated with poorer survival, whereas overweight and mild obesity are frequently associated with neutral to modestly protective effects on OS (2, 19, 25, 34). Selected series have reported adverse associations of obesity with oncologic endpoints in specific subsites or stage contexts, underlining the setting-dependent nature of BMI–outcome associations (21, 23). Iyengar et al. found obesity to be an independent adverse prognostic factor in early-stage (T1–T2) oral tongue squamous cell carcinoma, a cohort deliberately restricted to minimize tumor-related weight loss (21). Wang et al. similarly reported worse outcomes in obese compared with normal-weight OSCC patients in a large northern Chinese cohort across all stages; however, the authors themselves acknowledged that the applicability of WHO BMI cutoffs to Chinese populations remains uncertain, limiting direct comparability with European cohorts (23). In contrast, our Central European cohort covers the full spectrum of T-stages. Although underweight patients were relatively few, they disproportionately presented with advanced disease characteristics, including higher rates of pT4 tumors, nodal positivity, and lymphovascular invasion. This suggests the survival gradient may partly reflect nutritional depletion in underweight patients rather than a protective effect of obesity per se.

In our adjusted models, obesity was associated with significantly improved OS compared with normal weight. When modeled continuously, higher BMI independently predicted better OS. The non-significant overall association of BMI category with OS may reflect limited precision across BMI categories, particularly given the small number of underweight patients. Categorizing continuous predictors is known to reduce statistical power and may distort effect estimates (35, 36). For RFS, no significant association with BMI was observed after adjustment, although the continuous BMI estimate suggested a non-significant protective trend, while nodal positivity was a significant predictor of recurrence risk, consistent with previous reports identifying nodal disease as a key clinicopathological predictor of recurrence in OSCC (37). Reverse causation is an potential consideration in BMI–mortality research, particularly in OSCC where tumor-related dysphagia, odynophagia, pain, and reduced intake may cause pretreatment weight loss and malnutrition (38). In our cohort, underweight patients more frequently presented with advanced clinicopathological features, which may partly explain the observed survival gradient. Nevertheless, the association of higher BMI with improved OS persisted after adjustment for major prognostic factors, suggesting that BMI may capture aspects of host reserve beyond stage alone and may serve as a pragmatic marker to support risk stratification and early supportive-care planning. These findings are broadly consistent with the European literature on OSCC survival: Richter et al., in a German single-center OSCC cohort, reported independently reduced OS risk for normal-weight (HR 0.32) and overweight patients (HR 0.17) compared with underweight. For RFS, Richter et al. additionally observed a significantly reduced recurrence risk in overweight patients (HR 0.28, p = 0.031), a stronger effect than the non-significant trend observed in our cohort, potentially reflecting differences in cohort composition or statistical power (25). Chang et al. similarly identified underweight as an independent adverse prognostic factor for OS in a Taiwanese surgical cohort (2).

In contrast to most OSCC studies focusing on BMI and OS/RFS, our study additionally assessed key perioperative endpoints. Hospital LOS and PEG placement were not independently associated with BMI after adjustment, while findings for ICU LOS depended on BMI modeling. Across these perioperative outcomes, free-flap reconstruction and tumor burden were important determinants. This is consistent with evidence that postoperative pathways, operative complexity, and disease extent strongly influence LOS and ICU use in head and neck oncologic surgery (39–41). For PEG placement, our findings support an individualized, stage- and complexity-driven approach: PEG decisions are typically driven by anticipated functional impairment, tumor site/stage, and treatment intensity rather than baseline anthropometrics (42–45). Accordingly, patients with advanced disease and anticipated clinically relevant dysphagia or inadequate oral intake should receive early nutritional assessment and, when indicated, timely enteral nutrition via nasogastric or percutaneous routes, consistent with guideline-based supportive care in head and neck cancer (27, 46). At the same time, routine prophylactic PEG placement is not recommended, as a relevant proportion of tubes may remain unused and procedure-related morbidity can occur (47).

In contrast, transfusion-related outcomes showed a consistent inverse association with BMI. Higher BMI was independently associated with lower odds of RBC transfusion and of greater transfusion burden, while categorical modeling showed significantly lower odds for both outcomes in patients with obesity. Similar signals have been reported in major head and neck and free-flap cohorts, in which low BMI/underweight has been linked to higher transfusion risk (48–50). Low BMI may reflect reduced physiologic reserve and vulnerability to perioperative blood loss or anemia; however, transfusion practice is also influenced by baseline hemoglobin, intraoperative blood loss, operative time, and institutional thresholds, which were not comprehensively captured here (50–52). Given that recent studies indicate shorter OS and RFS among patients receiving perioperative RBC transfusions during primary OSCC surgery, these results are of clinical relevance (53–55). Across models, free-flap reconstruction remained the strongest predictor, consistent with prior work showing transfusion needs are largely driven by procedural complexity while patient factors provide incremental information (53, 54, 56, 57).

The retrospective single-center design limits generalizability and leaves residual confounding possible. BMI is an imperfect proxy for nutritional status, and the lack of body-composition measures, weight-loss trajectories, and frailty/functional markers restricts mechanistic interpretation and increases susceptibility to reverse causation (33, 58–60). The small number of underweight patients and pooling of obesity classes reduced precision, and continuous modeling assumes linearity. Finally, the complete-case approach may introduce selection effects. Clinically, pretreatment BMI may still help identify patients at higher vulnerability—particularly those with low BMI—for early nutritional assessment and structured supportive-care planning (2, 25).

## 5 Conclusion

In this retrospective single-center cohort of primary, curative-intent R0-resected OSCC, pretreatment BMI was significantly associated with improved OS (HR per 1 kg/m² 0.95, 95% CI 0.91–0.99). For RFS, BMI showed only a non-significant protective trend after adjustment. Regarding perioperative endpoints, BMI was significantly associated with fewer RBC transfusions. Nevertheless, PEG placement and hospital LOS were not independently related to BMI, while findings for ICU LOS depended on BMI modeling. Across these perioperative outcomes, surgical complexity and tumor burden remained important determinants. Given the retrospective design and residual confounding, these findings must be interpreted with caution; pretreatment BMI may help identify vulnerable low-BMI patients for early nutritional assessment and supportive-care planning, and prospective multicenter studies incorporating weight trajectories and body composition are warranted.

## Data Availability

The datasets presented in this article are not readily available because of patient privacy and institutional data protection restrictions. Requests to access the datasets should be directed to EF, eike.floegel@uniklinik-freiburg.de.

## Author’s note

Parts of the results of this study were presented at the 28th EACMFS Congress (15–18 September 2026, Athens, Greece) and were published in abstract form in the *Journal of Cranio-Maxillofacial Surgery* as: “Pretreatment body mass index as a prognostic factor for clinical course and survival in primary surgically treated oral squamous cell carcinoma – a retrospective study,” *Journal of Cranio-Maxillofacial Surgery*, Volume 54, Supplement 1, 2026, 107611. https://doi.org/10.1016/j.jcms.2026.107611.
