# Supplementary material for: Pretreatment body mass index as a prognostic factor for clinical course and survival in primary surgically treated oral squamous cell carcinoma – a retrospective study
Source: Front Oncol. 2026 Oct 2;16:1894543. doi: 10.3389/fonc.2026.1894543 (PMC13671965; doi:10.3389/fonc.2026.1894543)
Supplement: Supplementary file 1 [file DataSheet1.pdf]

Supplementary Material

1 Supplementary Tables

1.1 **Supplementary Table S1.** Univariable associations with overall survival and recurrence-free survival

	**OS**			**RFS**		
	HR	95% CI	P-value	HR	95% CI	P-value
**BMI**						
Normal weight	1			1		
Underweight	1.96	1.03–3.77	0.042	1.62	0.89–2.95	0.112
Overweight	1.14	0.76–1.73	0.526	1.12	0.76–1.64	0.558
Obesity	0.55	0.30–1.04	0.065	0.69	0.41–1.17	0.166
**BMI per +1 kg/m²**	0.95	0.92–0.99	0.015	0.97	0.93–1.00	0.053
**Sex**						
Male	1			1		
Female	1.19	0.82–1.72	0.357	1.06	0.76–1.48	0.725
**Age**						
per +1-year	1.02	1.00–1.04	0.015	1.01	1.00–1.03	0.053
**Grading**						
G1	1			1		
G2	2.18	0.80–5.94	0.128	1.31	0.61–2.82	0.493
G3-4	2.74	0.95–7.86	0.062	1.70	0.75–3.87	0.207

**pT Stage**						
T1	1			1		
T2	2.08	1.18–3.68	0.012	1.77	1.11–2.83	0.017
T3	3.93	2.18–7.10	<0.001	2.29	1.37–3.82	0.002
T4	3.85	2.14–6.91	<0.001	2.29	1.37–3.81	0.001
**pN Stage**						
N0	1			1		
N1	1.49	0.89–2.49	0.132	1.38	0.87–2.21	0.170
N2	2.35	1.48–3.73	<0.001	2.00	1.30–3.09	0.002
N3	3.57	1.82–7.00	<0.001	2.83	1.50–5.33	0.001
**pN Stage (binary)**						
N0	1			1		
N+	2.06	1.43–2.97	<0.001	1.80	1.28–2.51	<0.001
**LVI**						
No	1			1		
Yes	1.98	1.35–2.90	<0.001	1.74	1.22–2.46	0.002
**Alcohol**						
No	1			1		
Yes	1.12	0.59–2.15	0.729	1.39	0.80–2.42	0.239
**Nicotine**						
No	1			1		
Yes	0.85	0.56–1.30	0.460	0.83	0.57–1.22	0.343

**Alcohol or Nicotine**						
None	1			1		
Alcohol	1.20	0.60–2.40	0.612	1.51	0.83–2.76	0.180
Nicotine	0.96	0.60–1.56	0.866	0.98	0.63–1.52	0.915
Alcohol + Nicotine	1.26	0.80–1.96	0.316	1.30	0.86–1.97	0.214
**Diabetes mellitus**						
No	1			1		
Yes	1.79	1.16–2.76	0.008	1.49	0.99–2.23	0.054
**Free-flap Reconstruction**						
No	1			1		
Yes	2.06	1.39–3.05	<0.001	1.76	1.24–2.50	0.002
**Hospital length of stay (days)**						
per +1 day	1.01	1.00–1.02	0.010	1.01	1.00–1.02	0.191
**HPV**						
No	1			1		
Yes	0.94	0.77–1.15	0.551	0.92	0.76–1.10	0.337
**PEG**						
No	1			1		
Yes	2.23	1.47–3.39	<0.001	1.64	1.10–2.44	0.016

Univariable Cox proportional hazards models for overall survival (OS) and recurrence-free survival (RFS) are presented as hazard ratios (HRs) with 95% confidence intervals (CIs) and two-sided P-values (Wald test). BMI is shown categorically (reference: normal weight) and continuously (per +1 kg/m²). Reference categories are indicated as HR = 1. Abbreviations: OS, overall survival; RFS, recurrence-free survival; HR, hazard ratio; CI, confidence interval; BMI, body mass index; LVI, lymphovascular invasion; HPV, human papillomavirus; PEG, percutaneous endoscopic gastrostomy.

1.2 **Supplementary Table S2.** Multivariable Poisson regression analyses of hospital and ICU length of stay with pretreatment BMI modeled categorically and continuously.

**BMI modeled categorically**	**Hospital LOS**			**ICU LOS**		
	IRR	95% CI	P-value	IRR	95% CI	P-value
**BMI**						
Normal weight	1			1		
Underweight	1.01	0.90–1.12	0.911	1.26	0.97–1.63	0.079
Overweight	0.97	0.91–1.04	0.374	0.90	0.77–1.06	0.204
Obesity	0.99	0.91–1.07	0.728	1.13	0.94–1.37	0.184
**Sex**						
Male	1			1		
Female	0.93	0.88–0.99	0.016	1.26	1.09–1.45	0.001
**Age**						
per 1-year increase	1.00	1.00–1.01	<0.001	1.01	1.00–1.02	0.002
**pT Stage**						
T1	1			1		
T2	1.17	1.07–1.27	0.001	1.15	0.91–1.45	0.245
T3	1.40	1.27–1.55	<0.001	1.17	0.91–1.51	0.221
T4	1.58	1.44–1.75	<0.001	1.34	1.05–1.72	0.021
**pN Stage (binary)**						
N0	1			1		
N+	1.14	1.08–1.21	<0.001	1.08	0.94–1.24	0.289

**Free-flap Reconstruction**						
No	1			1		
Yes	1.59	1.48–1.71	<0.001	2.88	2.37–3.50	<0.001
**Diabetes mellitus**						
No	1			1		
Yes	1.05	0.97–1.12	0.211	1.21	1.03–1.42	0.024

**BMI modeled continuously**	Hospital LOS			ICU LOS		
	IRR	95% CI	P-value	IRR	95% CI	P-value
**BMI**						
BMI per +1 kg/m²	1.00	0.99–1.00	0.345	0.99	0.98–1.01	0.460
**Sex**						
Male	1			1		
Female	0.93	0.88–0.99	0.013	1.23	1.07–1.41	0.003
**Age**						
per 1-year increase	1.00	1.00–1.01	<0.001	1.01	1.00–1.02	0.003
**pT Stage**						
T1	1			1		
T2	1.17	1.07–1.27	0.001	1.17	0.92–1.47	0.194
T3	1.40	1.27–1.55	<0.001	1.20	0.93–1.54	0.163
T4	1.58	1.43–1.74	<0.001	1.39	1.09–1.78	0.008
**pN Stage (binary)**						

N0	1			1		
N+	1.14	1.08–1.21	<0.001	1.09	0.95–1.25	0.214
**Free-flap Reconstruction**						
No	1			1		
Yes	1.59	1.49–1.71	<0.001	2.79	2.30–3.37	<0.001
**Diabetes mellitus**						
No	1			1		
Yes	1.05	0.98–1.13	0.182	1.24	1.05–1.46	0.010

Multivariable Poisson regression models for hospital length of stay (LOS) and ICU LOS are presented as incidence rate ratios (IRRs) with 95% confidence intervals (CIs) and two-sided P-values (Wald test). BMI is shown categorically (WHO categories; reference: normal weight) and continuously (per +1 kg/m²), as indicated. ICU LOS was analyzed among ICU-admitted patients only. All models were adjusted for sex, age at surgery, pT stage, pN stage (binary), free-flap reconstruction, and diabetes mellitus. Reference categories are indicated as IRR = 1. Abbreviations: LOS, length of stay; ICU, intensive care unit; IRR, incidence rate ratio; CI, confidence interval; BMI, body mass index.

1.3 **Supplementary Table S3.** Multivariable logistic and ordinal logistic regression analyses of perioperative red blood cell transfusion outcomes with pretreatment BMI modeled categorically and continuously.

**BMI modeled categorically**		**RBC transfusion**			**Number of RBC units transfused**	
	OR	95% CI	P-value	OR	95% CI	P-value
**BMI**						
Normal weight	1			1		
Underweight	0.66	0.14–3.05	0.591	0.98	0.26–3.74	0.979
Overweight	0.62	0.28–1.33	0.218	0.57	0.28–1.14	0.114
Obesity	0.18	0.06–0.57	0.004	0.17	0.06–0.53	0.002
**Sex**						
Male	1			1		
Female	1.18	0.58–2.41	0.642	1.27	0.66–2.44	0.465
**Age**						
per 1-year increase	1.01	0.98–1.04	0.718	1.01	0.99–1.04	0.294
**pT Stage**						
T1	1			1		
T2	2.09	0.57–7.63	0.265	1.90	0.54–6.71	0.321
T3	2.50	0.65–9.63	0.183	2.44	0.66–9.01	0.181
T4	4.38	1.15–16.63	0.030	3.36	0.94–12.05	0.063
**pN Stage (binary)**						
N0	1			1		
N+	2.75	1.37–5.53	0.005	2.66	1.41–5.00	0.002

**Free-flap Reconstruction**						
No	1			1		
Yes	37.20	8.43–164.26	<0.001	43.41	9.78–192.62	<0.001
**Diabetes mellitus**						
No	1			1		
Yes	2.00	0.80–5.02	0.139	2.95	1.27–6.84	0.012

**BMI modeled continuously**		RBC transfusion			**Number of RBC units transfused**	
	OR	95% CI	P-value	OR	95% CI	P-value
**BMI**						
BMI per +1 kg/m²	0.91	0.84–0.98	0.012	0.89	0.82–0.95	0.001
**Sex**						
Male	1			1		
Female	1.30	0.65–2.62	0.457	1.40	0.74–2.68	0.304
**Age**						
per 1-year increase	1.01	0.98–1.04	0.644	1.02	0.99–1.04	0.266
**pT Stage**						
T1	1			1		
T2	1.96	0.54–7.10	0.306	1.84	0.52–6.49	0.346
T3	2.18	0.57–8.31	0.253	2.18	0.59–8.05	0.240
T4	3.53	0.95–13.14	0.060	2.88	0.81–10.23	0.102
**pN Stage (binary)**						

N0	1			1		
N+	2.55	1.30–5.04	0.007	2.58	1.39–4.80	0.003
**Free-flap Reconstruction**						
No	1			1		
Yes	40.05	9.08–176.67	<0.001	46.96	10.60–208.12	<0.001
**Diabetes mellitus**						
No	1			1		
Yes	1.88	0.76–4.65	0.172	2.99	1.29–6.94	0.011

Multivariable models are presented as odds ratios (ORs) with 95% confidence intervals (CIs) and two-sided P-values (Wald test). Any RBC transfusion was analyzed using logistic regression; number of RBC units transfused was analyzed using ordinal logistic regression (proportional odds), with ORs representing the odds of a higher transfusion category. BMI is shown categorically (WHO categories; reference: normal weight) and continuously (per +1 kg/m^2), as indicated. All models were adjusted for sex, age at surgery, pT stage, pN stage (binary), free-flap reconstruction, and diabetes mellitus. Reference categories are indicated as OR = 1. Abbreviations: BMI, body mass index; CI, confidence interval; OR, odds ratio; RBC, red blood cell.

1.4 **Supplementary Table S4.** Multivariable logistic regression analyses of PEG placement with pretreatment BMI modeled categorically and continuously.

**BMI modeled categorically**	**PEG**		
	OR	95% CI	P-value
**BMI**			
Normal weight	1		
Underweight	2.57	0.64–10.28	0.181
Overweight	0.74	0.31–1.77	0.503
Obesity	1.30	0.45–3.79	0.627
**Sex**			
Male	1		
Female	0.85	0.40–1.84	0.682
**Age**			
per 1-year increase	1.02	0.99–1.06	0.163
**pT Stage**			
T1	1		
T2	2.67	0.30–23.42	0.376
T3	12.00	1.40–102.61	0.023
T4	19.32	2.29–162.86	0.006
**pN Stage (binary)**			
N0	1		
N+	1.50	0.72–3.11	0.283

**Free-flap Reconstruction**			
No	1		
Yes	7.22	2.14–24.39	0.001
**Diabetes mellitus**			
No	1		
Yes	0.37	0.13–1.04	0.059

**BMI modeled continuously**	PEG		
	OR	95% CI	P-value
**BMI**			
BMI per +1 kg/m²	0.95	0.88–1.03	0.197
**Sex**			
Male	1		
Female	0.80	0.38–1.70	0.563
**Age**			
per 1-year increase	1.02	0.99–1.06	0.156
**pT Stage**			
T1	1		
T2	2.81	0.32–24.60	0.350
T3	12.93	1.52–109.99	0.019
T4	20.89	2.51–173.49	0.005
**pN Stage (binary)**			

N0	1		
N+	1.52	0.74–3.13	0.259
**Free-flap Reconstruction**			
No	1		
Yes	6.49	2.01–20.96	0.002
**Diabetes mellitus**			
No	1		
Yes	0.44	0.16–1.22	0.115

Multivariable logistic regression models for PEG placement are presented as odds ratios (ORs) with 95% confidence intervals (CIs) and two-sided P-values (Wald test). BMI is shown categorically (WHO categories; reference: normal weight) and continuously (per +1 kg/m²), as indicated. All models were adjusted for sex, age at surgery, pT stage, pN stage (binary), free-flap reconstruction, and diabetes mellitus. Reference categories are indicated as OR = 1. Abbreviations: PEG, percutaneous endoscopic gastrostomy; OR, odds ratio; CI, confidence interval; BMI, body mass index.

2 Supplementary Figures

2.1 **Supplementary Figure S1.** BMI distribution stratified by age and sex

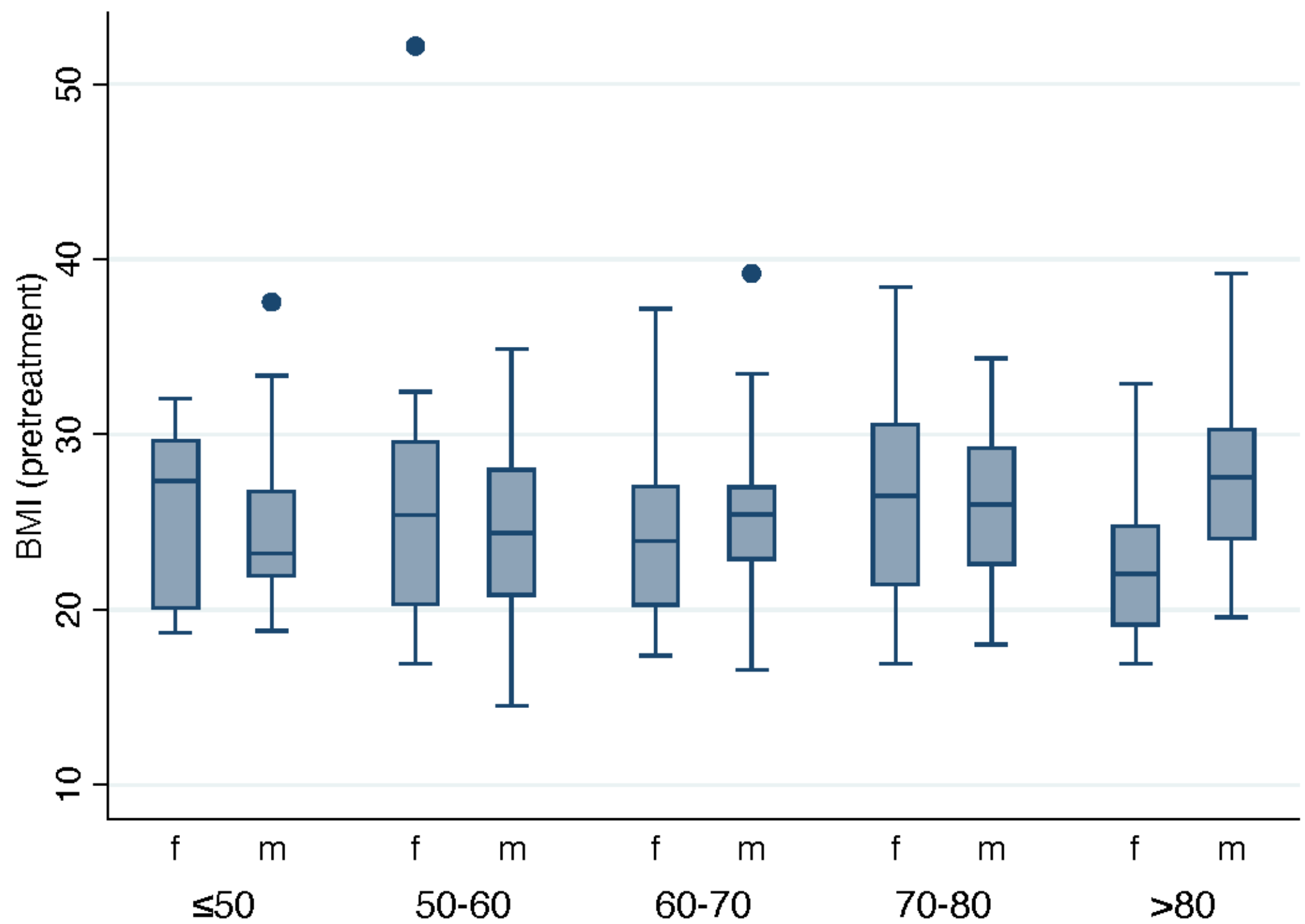

Boxplots showing pretreatment BMI (kg/m²) stratified by age at surgery and sex.

2.2 **Supplementary Figure S2.** Kaplan–Meier curves for overall survival stratified by age

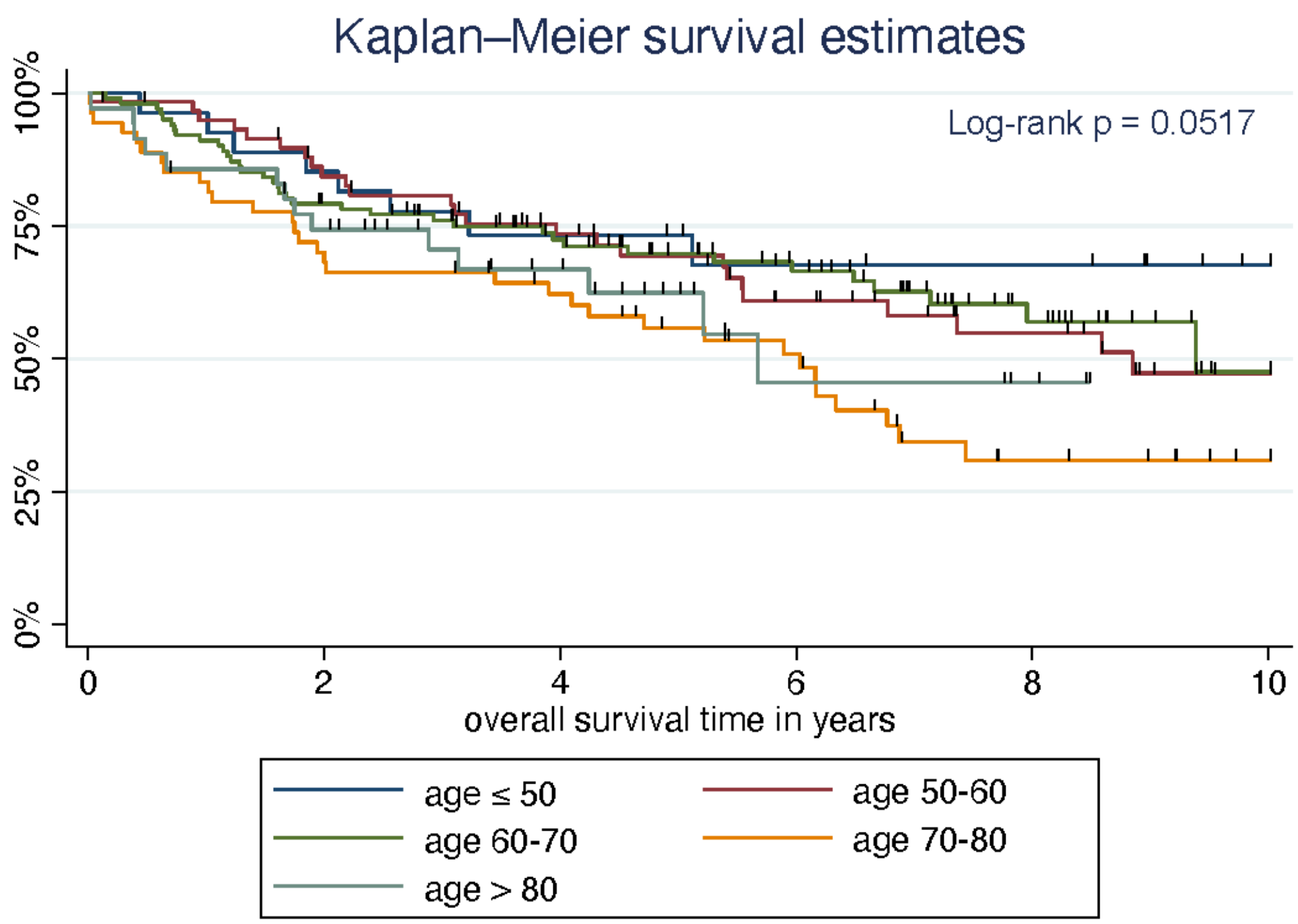

Kaplan–Meier estimates of overall survival stratified by age at surgery over 10 years of follow-up.

2.3 **Supplementary Figure S3.** Kaplan–Meier curves for overall survival stratified by pT stage

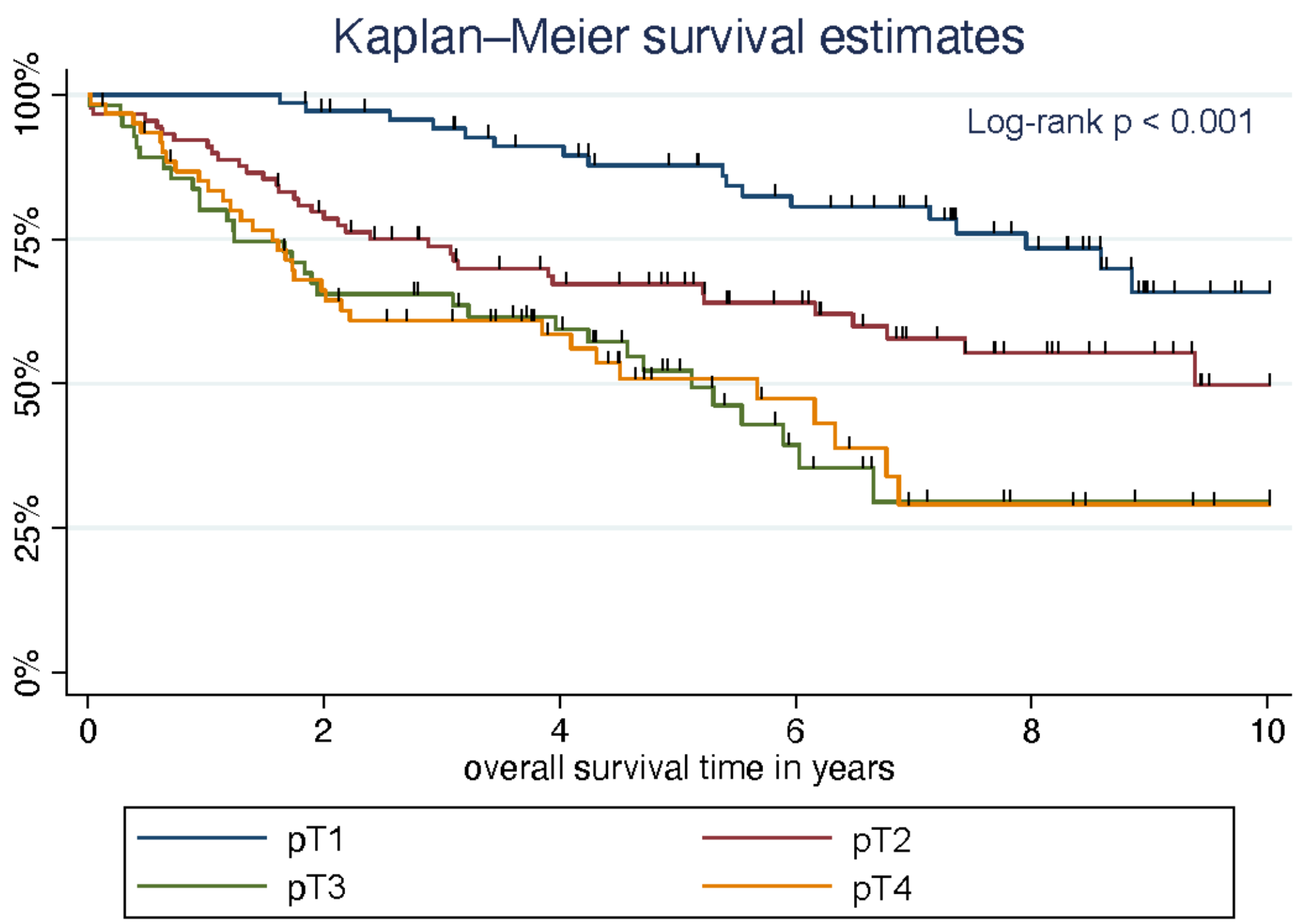

Kaplan–Meier estimates of overall survival stratified by pathological tumor stage over 10 years of follow-up.

2.4 **Supplementary Figure S4.** Kaplan–Meier curves for overall survival stratified by pN stage

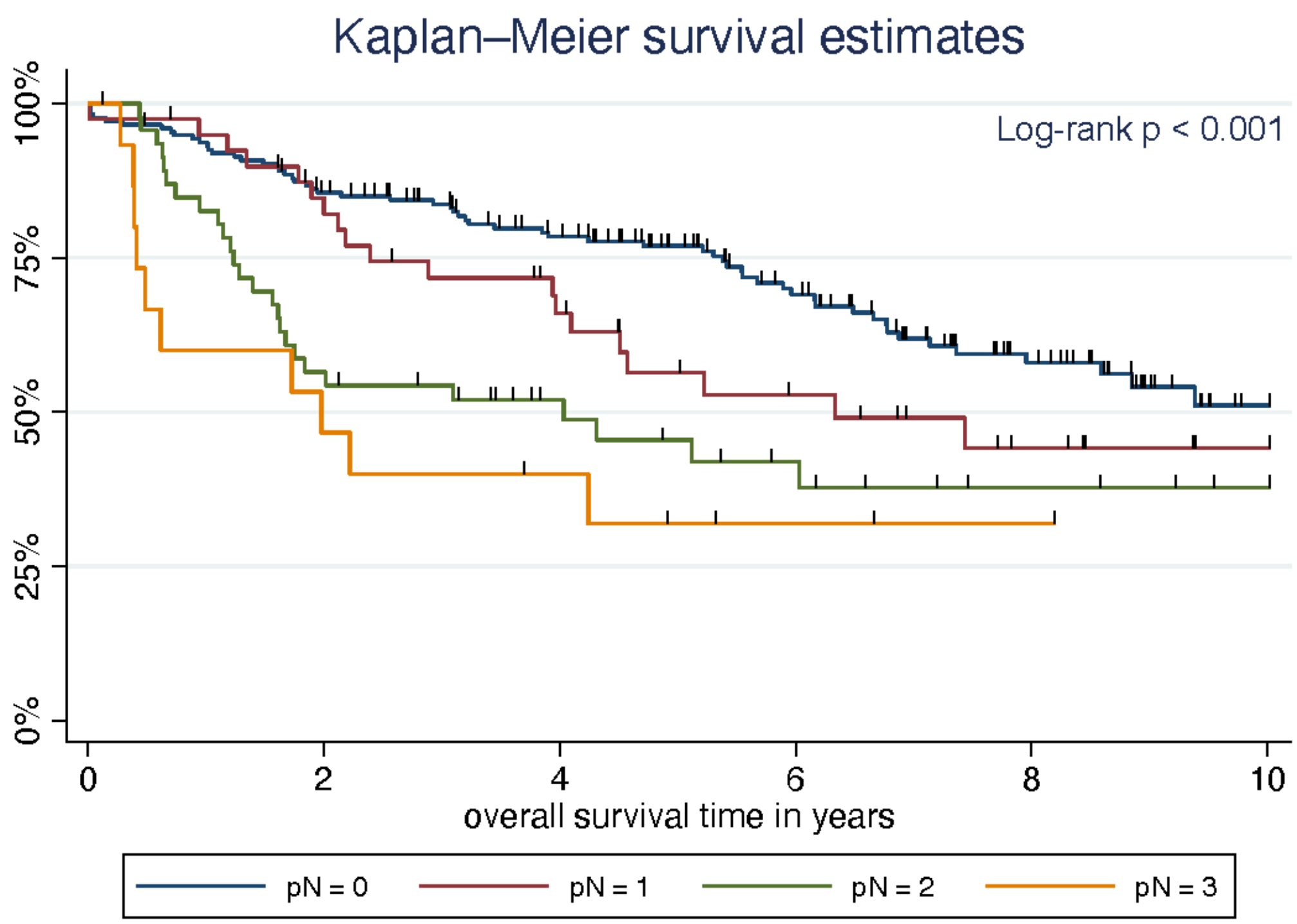

Kaplan–Meier estimates of overall survival stratified by pathological nodal stage over 10 years of follow-up.

2.5 **Supplementary Figure S5.** Kaplan–Meier curves for recurrence-free survival stratified by age

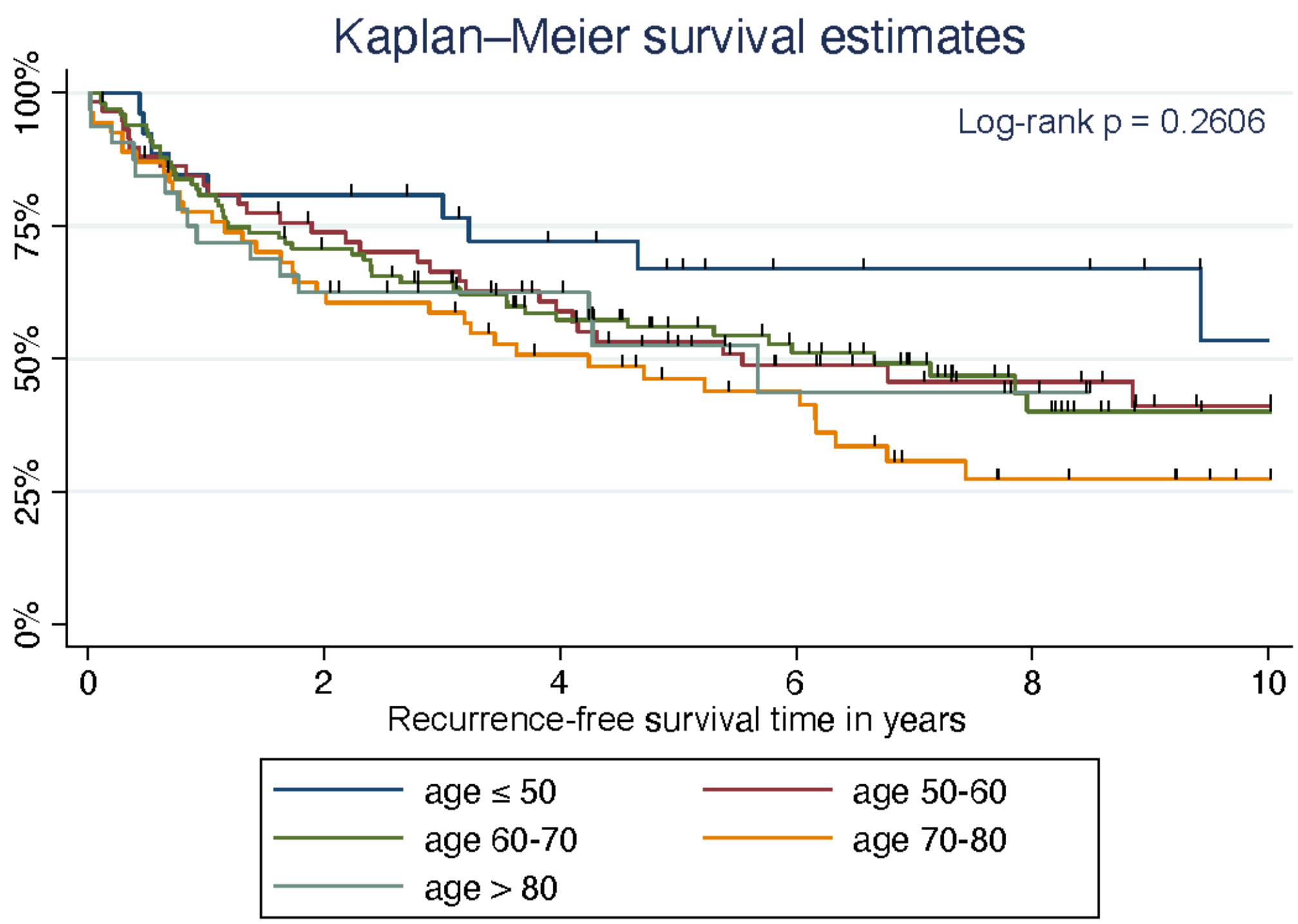

Kaplan–Meier estimates of recurrence-free survival stratified by age at surgery over 10 years of follow-up.

2.6 **Supplementary Figure S6.** Kaplan–Meier curves for recurrence-free survival stratified by pT stage

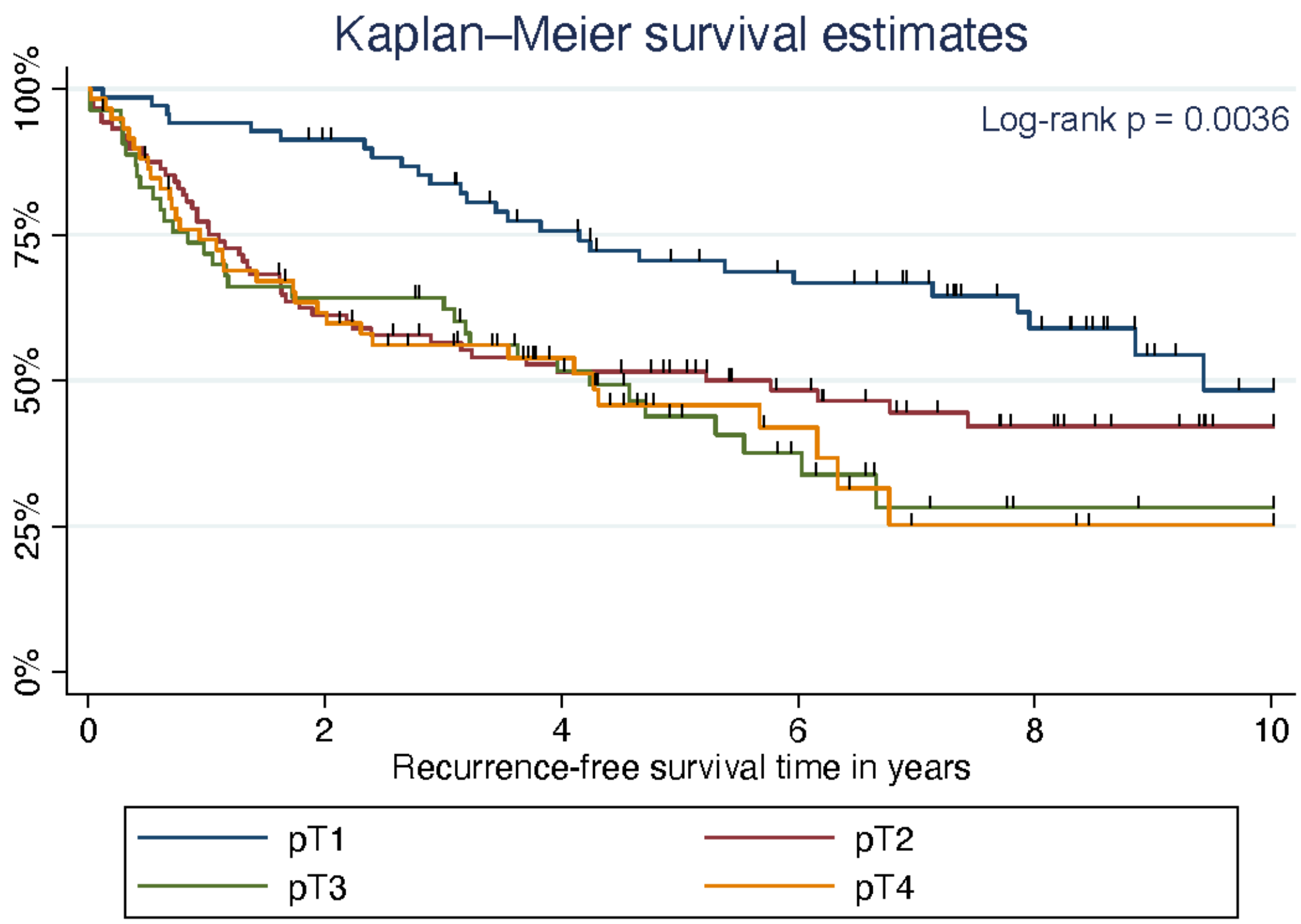

Kaplan–Meier estimates of recurrence-free survival stratified by pathological tumor stage over 10 years of follow-up.

2.7 **Supplementary Figure S7.** Kaplan–Meier curves for recurrence-free survival stratified by pN stage

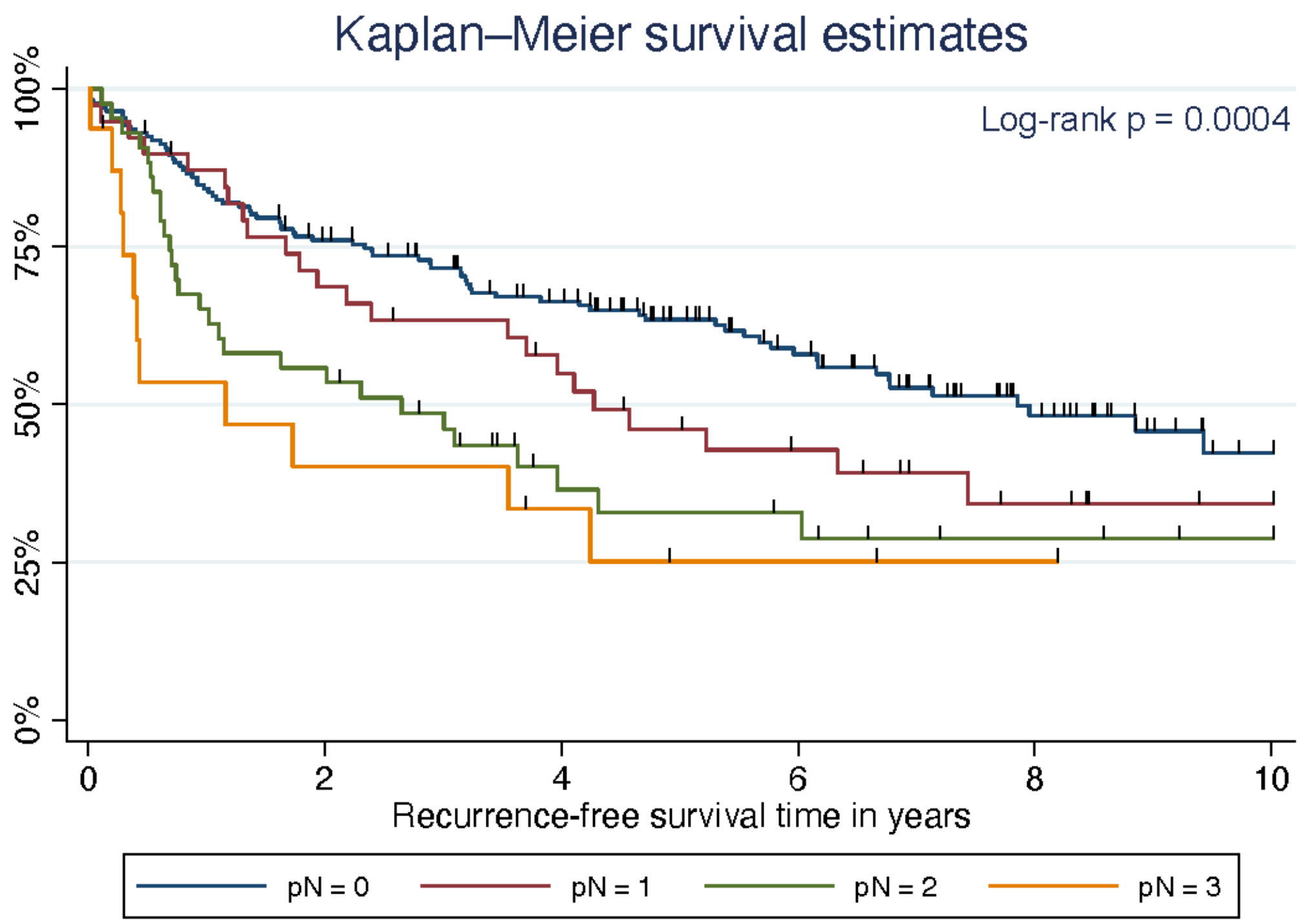

Kaplan–Meier estimates of recurrence-free survival stratified by pathological nodal stage over 10 years of follow-up.
